# Developmental Validation of DNA Quantitation System, Extended STR Typing Multiplex, and Database Solutions for *Panthera leo* Genotyping

**DOI:** 10.3390/life15040664

**Published:** 2025-04-17

**Authors:** Lenka Vankova, Johana Alaverdyan, Daniel Vanek

**Affiliations:** 1 Institute for Environmental Sciences, Charles University Prague, Benatska 2, 12800 Prague, Czech Republic; lenka.vankova@fdnas.cz (L.V.); johana.alaverdyan@gmail.com (J.A.); 2 Forensic DNA Service, Budinova 2, 18081 Prague, Czech Republic; 3 Bulovka University Hospital, Budinova 2, 18000 Prague, Czech Republic; 4 Department of Forensic Medicine, Second Faculty of Medicine, Charles University, 11000 Stare Mesto, Czech Republic

**Keywords:** DNA quantitation, species determination, STR multiplex, CITES organism, forensic genetics, databasing, matching analysis, wildlife

## Abstract

This study describes the development of a species determination/DNA quantification system called Pleo Qplex and an individual identification STR multiplex called Pleo STRplex using *Panthera leo* samples. Pleo Qplex enables us to measure the quantity of extracted nuclear and mitochondrial DNA and detect the presence of co-purified inhibitors. Pleo STRplex, consisting of seven loci, enables the determination of the DNA profile from a sample of *Panthera leo* based on the analysis of short tandem repeats (STRs). The Pleo STRplex provides additional loci on top of previously published STR loci in Ptig STRplex and contains a specific STR marker that confirms *Panthera leo*. An allelic ladder of all STR markers was prepared to enable reliable allele calling. The STR loci can also be used to type the DNA of other members of the genus Panthera. The work on the resulting STR profiles is performed using GenoProof Suite, which offers databasing, matching, and relationship analysis.

## 1. Introduction

Lions (*Panthera* spp.) are a nearly extinct species [1]. *Panthera leo* was recently assessed for The IUCN Red List of Threatened Species in 2023 [2], and is listed as Vulnerable under criteria A2abcd. Lions are hunted for sport [3,4], poached [5], used in traditional medicine [6,7], in an expensive wine also used in traditional medicine [8], or killed by farmers in retaliation for livestock attacks [9]. PCR or qPCR is considered the ‘gold standard’ in many scientific disciplines, including forensic DNA typing. Quantitation of DNA extracts is essential in forensic genetics workflow [10]. Detecting inhibitors in stain extracts is crucial for downstream identification analysis [11], so qPCR-based quantitation is the method of choice [12,13,14]. Short tandem repeats (STRs) [15] constitute the bulk of entries in forensic DNA databases [16,17], making them essential polymorphisms for DNA-based forensic identification. DNA typing of animals in forensic labs initially targeted species determination [18] and individual identification of domesticated animals [19,20,21], where the animal derivatives can help to connect victims and suspects [22]. STR profiling of animal species has broader applications, such as conservation genetics [23] or food safety [24]. Forensic genetics is used in cases of illegal wildlife trade and poaching of highly endangered species like rhinos [25,26], pangolins [27], tortoises [28,29], parrots [30,31], cranes [32] or elephants [33,34]. This technical note describes the procedures for developing genetic systems for species determination/DNA quantification (*Pleo Qplex*) and individual identification (*Pleo STRplex*) of *Panthera leo* samples. Similar systems have been created for DNA quantification and multiplex STR analysis of *Panthera tigris* samples [35]. The STR loci can be used for DNA typing of other members of genus Panthera (*Panthera tigris*, *Panthera leo*, *Panthera pardus*, *Panthera onca*, *Panthera uncia*). Developing a set of STR loci that can be applied simultaneously to multiple species is consistent with previous efforts [36,37].

## 2. Material and Methods

### 2.1. Specimen Collection

The samples of the Panthera genus individuals were obtained through the Czech Environmental Inspectorate (Na Brehu 1a, 190 00 Prague, CZE) and various zoological gardens, submitted as an exhibit by the Czech Police, and from AAP (Animal Advocacy and Protection), a European animal welfare organization. The total number of tested *Panthera leo* individuals was 55 (23 hair samples, 1 fecal sample, 1 tanned hide, 20 blood samples, 4 skeletal/teeth samples, 2 tissue samples, and 4 claws), with 20 females and 36 males. The positive control was prepared from the tissue sample and was used for all sensitivity experiments. Other samples used for qPCR validation (100 samples of *P. tigris*, *P. pardus*, *P. uncia*, and *P. onca*) also came from the above specified sources. The sampling did not involve the infliction of trauma to living animals, as the samples came from dead animals. Therefore, this research was not subject to Directive 2010/63/EU of the European Parliament and Council [38], dated 22 September 2010, concerning the protection of animals used for scientific purposes.

### 2.2. DNA Extraction

DNA from hair, blood, and tissue samples was extracted using the Quick-DNA Micro-prep/Miniprep Plus Kit (Zymo Research, Irvine, CA, USA), while DNA from fecal samples was extracted using the Quick-DNA Fecal/Soil Microbe Miniprep Kit (Zymo Research, Irvine, CA USA). The protocol for the tanned hide was modified as described by Hebestreitova [39]. Teeth, bone, and claw samples were powdered using a SPEX cryogenic mill (SPEX, Los Angeles, CA, USA). DNA was then extracted with the PrepFiler^®^ BTA Forensic DNA Extraction Kit (Thermo Fisher Scientific, Sunnyvale, CA, USA) and purified using the OneStep PCR Inhibitor Removal Kit (Zymo Research, Irvine, CA, USA).

### 2.3. Pleo Qplex DNA Quantitation System

Pleo Qplex, a molecular system for species determination and DNA quantification, utilizes quantitative PCR (qPCR) with TaqMan probes, nuclear DNA primers, and primers highly specific for *Panthera leo* mtDNA. The assay is conducted in a single tube or well reaction.

Real-time reaction monitoring, including the detection of potential inhibition, was conducted using internal amplification of internal positive control (IPC) DNA (Forensic DNA Service, CZE). IPC DNA, an artificially prepared sequence of 366 oligonucleotides, prevents false negative results. Nuclear DNA concentrations were measured using the STR locus Pati01 [40]. These primers are not *Panthera leo* specific but amplify other big cats’ DNA. The concentration of mitochondrial DNA was measured using primers and probes targeted to the Cytochrome B gene of *Panthera leo* mtDNA. The target sequence is specific for *Panthera leo* only, with no observed mtDNA detection for other members of the genus Panthera (*P. tigris*, *P. pardus*, *P. uncia*, *P. onca*) or a tiger/lion hybrid (see Results/specificity study). The authors of this study donated human DNA used for the specificity tests. The composition of primers and probes in the *Pleo Qplex* quantitation mix is shown in Table 1.

### 2.4. qPCR Reaction Set-Up

The qPCR thermocycler QuantStudio 5 (Thermo Fisher Scientific, Sunnyvale, CA, USA) was used in this study. The qPCR conditions included an initial denaturation at 95 °C, followed by 50 cycles of 95 °C for 10 s and 60 °C for 20 s. The qPCR reaction mixture included 2× TaqMan Multiplex Master Mix (Thermo Fisher Scientific, Sunnyvale, CA, USA) and 20× qPleo Assay Mix for mtDNA, nuclear DNA, and IPC DNA (Forensic DNA Service, Prague, CZE). The final reaction volume was 10 µL, prepared as detailed in Table 2.

### 2.5. Pleo STRplex DNA Profiling System

The *Pleo STRplex* molecular system allows for the determination of the DNA profile of *Panthera leo* by analyzing short tandem repeats (STRs). The STRs in this multiplex include di-, tetranucleotide, or complex repetitions (refer to Table 3 and Table 4). The number of repeats within loci varies significantly, except for the locus Pleo32, which is monomorphic for *Panthera leo* but polymorphic for other big cats, and thus can serve as an internal control for species (*P. leo)* determination. Fluorescently labeled amplified STRs were then analyzed using capillary electrophoresis.

*Pleo STRplex* contains 7 pairs of fluorescently labeled primers. These primers have similar melting points and do not form primer dimers when used in the multiplex. The multiplex was optimized for 10 pg nDNA input. Positive and negative amplification controls were used throughout the validation process.

### 2.6. PCR Set-Up

The PCR mix consists of Gold Star 10× buffer (Promega, Madicon, WI, USA), AmpliTaq Gold DNA Polymerase (Thermo Fisher Scientific, USA), and 10× Pleo STRPlex Primer Mix (Forensic DNA Service, CZE). The total volume of the PCR reaction is 12.5 µL. For more details, refer to Table 5.

The PCR thermocycler MasterCycler Nexus gradient (Eppendorf, Hamburg, Germany) was used in this study. The PCR conditions were as follows: initial denaturation at 95 °C for 11 min, followed by 32 cycles of 94 °C for 30 s, 60 °C for 1 min and 10 s, and 72 °C for 1 min and 10 s. The final extension was at 72 °C for 60 min, followed by 60 °C for 60 min.

### 2.7. Fragment Analysis Set-Up

STR multiplex *Pleo STRPlex*: 1 µL PCR product + 12 µL deionized Hi-Di formamide (Thermo Fisher Scientific, USA) + 0.4 µL size standard LIZ600 (Thermo Fisher Scientific, Sunnyvale, CA, USA)

This study used the SeqStudio 3200 Genetic Analyzer (Thermo Fisher Scientific, Sunnyvale, CA, USA) for capillary electrophoresis. Raw data processing was performed using GeneMapper5 software (Thermo Fisher Scientific, Sunnyvale, CA, USA). Alleles of each STR marker were amplified using singleplex PCRs and combined into an allelic ladder. Alleles were also sequenced for verification.

### 2.8. Databasing

All resulting DNA profiles were stored in an XLS database in .csv format and subsequently uploaded into a matching database of Genoproof Suite, version 1.0.2 R629 (Qualitype, Dresden, Germany). Before use, it was necessary to customize the Genoproof Suite for animal samples. The customization steps were as follows: definition of the markers and the expected alleles, definition of the test kits, and uploading of the population data (if it exists). Genoproof Suite’s search function enables comparisons of the target profile with profiles in the matching database. The comparison setup enables the definition of stringency according to the ENFSI DNA WG recommendations for DNA Database Management [40]. The search preferences allow the number of mismatches and the minimal number of markers for a match to be set.

## 3. Results

### 3.1. qPCR Assay Pleo Qplex

The qPCR TaqMan assay meets the following criteria: r2 greater than 0.98 (linearity), efficiency between 89 and 108%, and standard deviations below 0.5 C_t_ (reproducibility). The lowest mitochondrial copy number providing positive *P. leo* determination was around 10 cp/rxn. The species specificity of qPCR Pleo Qplex assay was tested on 100 samples of other cats (*P. tigris*, *P. pardus*, *P. uncia*, and *P. onca*), and none of those provided positive results for mtDNA *P. leo*-specific target. Assays with DNA extracts from the blood, tissue, bone, teeth, and claw samples showed no inhibition during the qPCR. However, qPCR plots for several samples showed some inhibition, especially for hair and fecal samples. The OneStep PCR Inhibitor Removal Kit (Zymo Research, Irvine, CA, USA) successfully removed inhibitory substances.

**Figure 1:**
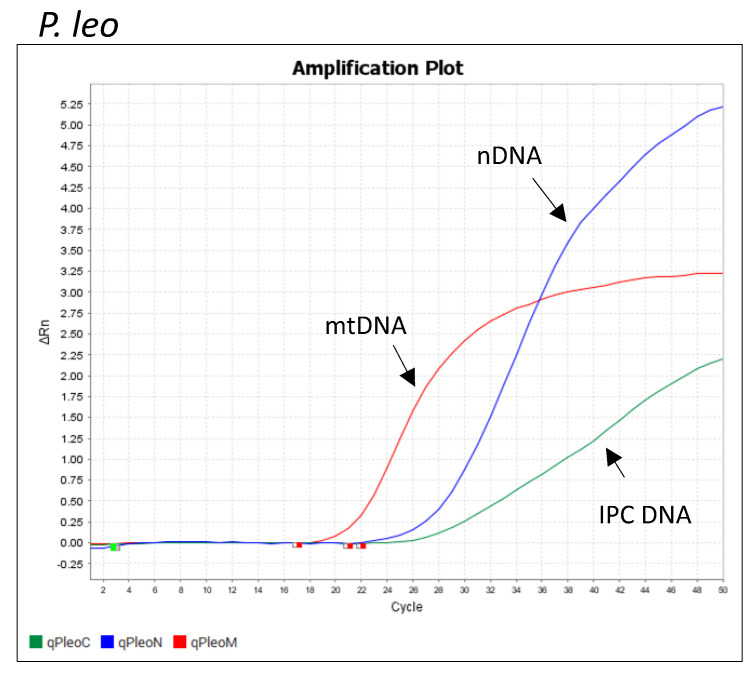
The qPCR plot generated using Pleo Qplex on a *Panthera leo* sample shows nuclear DNA quantitation (blue curve) and mitochondrial DNA (red curve). IPC DNA (green curve) is an internal positive control for qPCR.

**Figure 2:**
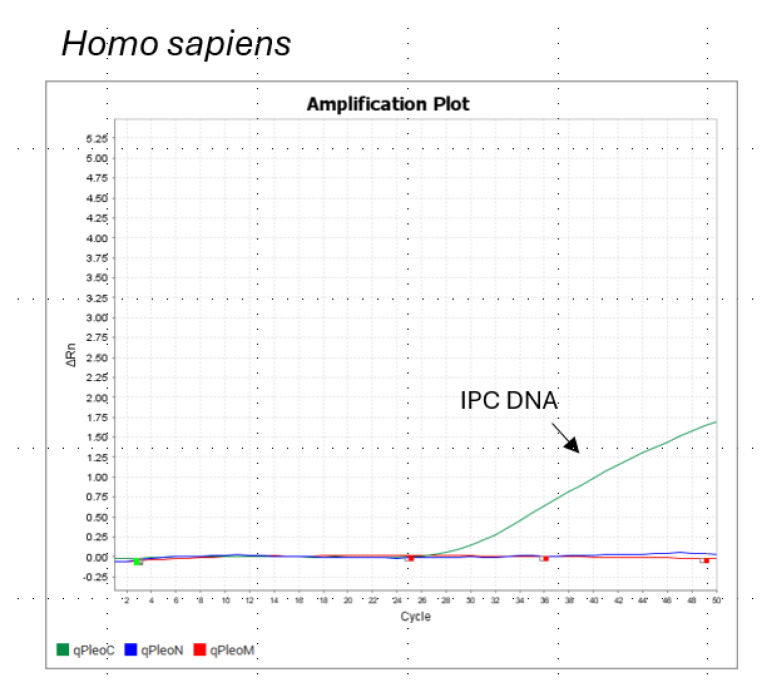
The qPCR plot generated using Pleo Qplex on a *Homo sapiens* sample shows nuclear DNA quantitation (blue curve) and mitochondrial DNA (red curve). IPC DNA (green curve) is an internal positive control for qPCR.

**Figure 3:**
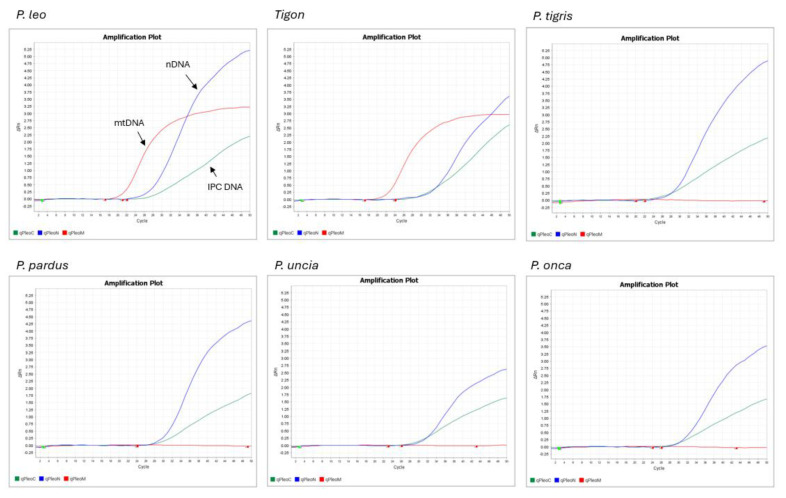
The qPCR plots generated using Pleo Qplex compare nuclear DNA quantitation (blue curve) and mitochondrial DNA (red curve) for various members of the genus Panthera (*P. tigris*, *P. pardus*, *P. uncia*, *P. onca*) and a tiger/lion hybrid. IPC DNA (green curve) is an internal positive control for qPCR.

Examples of QuantStudio 5 (Thermo Fisher Scientific, USA) quantitation and species determination outputs are shown in Figure 1, Figure 2 and Figure 3. Figure 1 shows the resulting qPCR plot generated using Pleo Qplex on the *Panthera leo* sample, Figure 2 shows the amplification plot for the sample of *Homo sapies*, and Figure 3 demonstrates the assay specificity for different members of the Panthera genus. Please note that the tiger/lion hybrid (tigon) can be falsely interpreted as a pure *P. leo* individual.

### 3.2. Pleo Qplex Specificity

The specificity of Pleo Qplex was tested on various members of the genus Panthera (*P. tigris*, *P. pardus*, *P. uncia*, *P. onca*) and a tiger/lion hybrid. The resulting qPCR plots (see Figure 3) clearly show that the mtDNA quantitation is specific for *P. leo* or Tigon only, while the nuclear marker provides quantitation results for all tested members of genus Panthera.

### 3.3. Pleo Qplex Sensitivity

**Figure 4:**
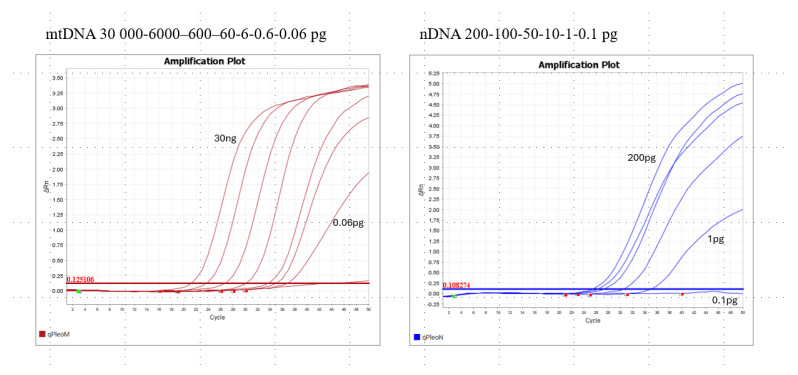
The results of Pleo Qplex sensitivity study.

The sensitivity test of Pleo Qplex was performed using serial dilutions of positive control DNA of *P. leo*. The plots in Figure 4 demonstrate the assay sensitivity for mitochondrial and nuclear DNA, with mtDNA being an order of magnitude more sensitive.

### 3.4. DNA Typing Pleo STRplex

**Figure 5:**
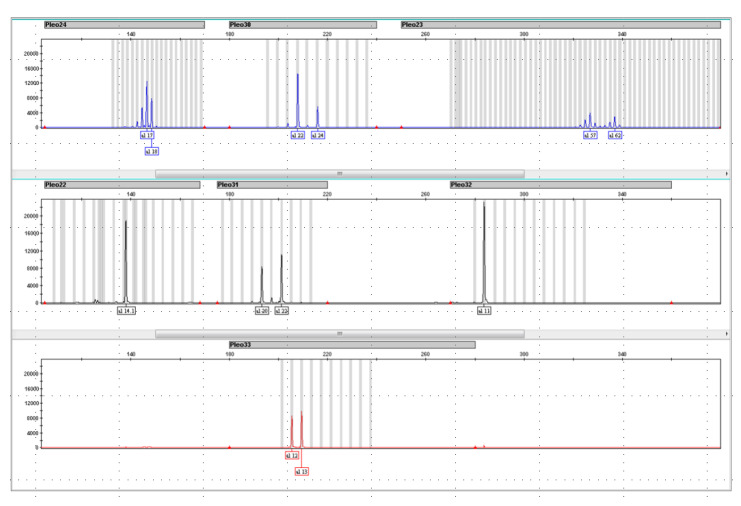
Resulting electropherogram (EPG) with a DNA profile consisting of 7 STRs from the *Panthera leo* positive control sample. The peak colors on the EPG (blue, black, red) reflect the fluorophore used for the 5′ primer fluorescent label (see Table 3).

**Figure 6:**
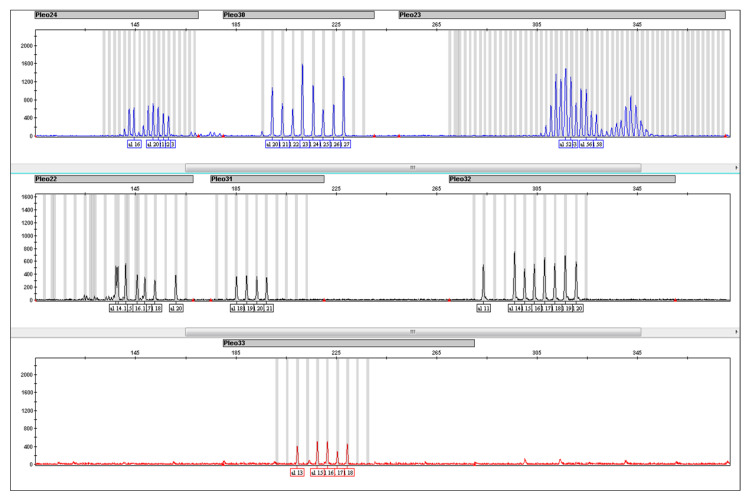
Resulting electropherogram (EPG) of the Pleo STRplex allelic ladder. This allelic was prepared to reflect the common alleles of other individuals belonging to the genus Panthera. The peak colors on the EPG (blue, black, red) reflect the fluorophore used for the 5’ primer fluorescent label (see Table 3).

**Figure 7:**
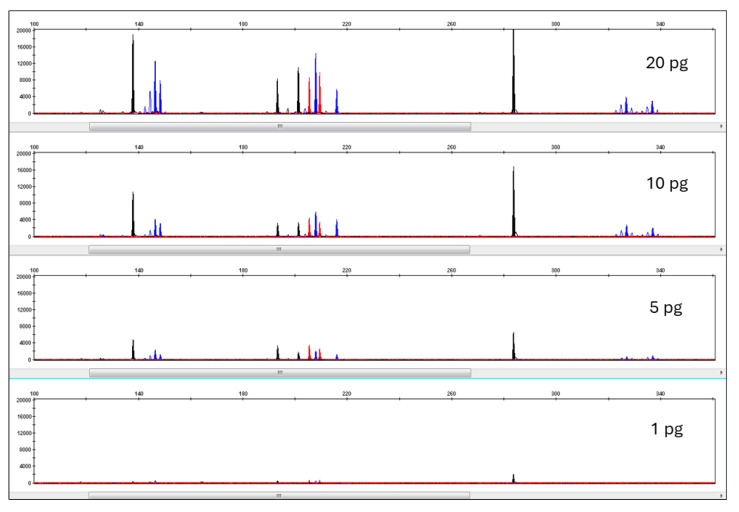
Resulting electropherograms of the Pleo STRplex with decreasing concentrations of input DNA extracted from *P. leo*. The peak colors on the EPG (blue, black, red) reflect the fluorophore used for the 5’ primer fluorescent label (see Table 3).

**Figure 8:**
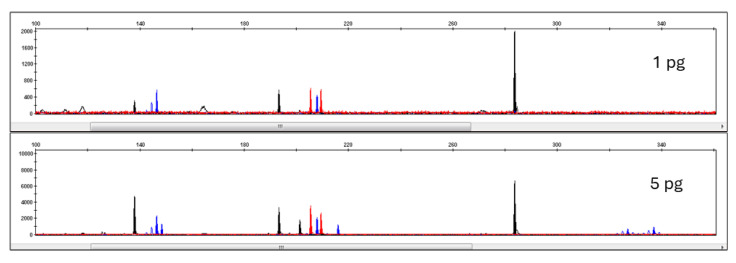
Reduction in peak height (5 pg) and allelic dropout (1 pg) in Pleo STRplex and other big cats. 5 pg plot was magnified 2×; 1 pg plot was magnified 10×. The peak colors on the EPG (blue, black, red) reflect the fluorophore used for the 5’ primer fluorescent label (see Table 3).

STR loci included in Pleo STRplex multiplex have 105–380 bp long amplicons. However, only two loci have amplicons longer than 240 bp. This multiplex is thus suitable even for low quantities of degraded DNA, as often observed in forensic samples. Figure 5 shows the resulting electropherogram of *a Panthera leo* individual, while the allelic ladder plot is shown in Figure 6. The individual alleles observed in different species of the genus Panthera are listed in Table 6. The sensitivity of Pleo STRplex was tested with decreasing amounts of nuclear DNA (See Figure 7 and Figure 8). The magnification of the DNA profile generated for 5 pg and 1 pg (Figure 8) shows a significant reduction in the peak height (5 pg) and allelic dropout (1 pg). The multiplex amplifies the DNA of other big cats. However, it is essential to point out that some of the alleles of other big cats are shifted outside the allele bins created for *Panthera leo*.

#### Pleo 32 Locus

STR locus Pleo 32 ((TCTG)n (TCTA)n, 4n, 270–360 bp), when tested on *P. leo* individuals (n = 55), always yielded only the allele 11. However, when other members of the genus Panthera were tested, we obtained a more polymorphic pattern (e.g., *P. pardus* (n = 14) yielded alleles 13, 14, 15, 16, 17; *P. tigris* (n = 76) yielded alleles 16, 17, 18, 19, 20, 21).

## 4. Discussion

DNA quantitation is essential in the forensic examination of biological/genetic evidence. Forensic DNA laboratories focused on samples of human origin adopted quantitative PCR as a golden standard [41]. Frequently used kits targeting human nuclear DNA (autosomes) and gonosomes (X- and Y-chromosomes) contain an internal positive control for detecting inhibitors [42,43,44], and also offer the possibility of measuring the degradation index [45,46]. Non-human DNA typing requires slightly different answers from the quantification step. The presence of male DNA is not an issue in animal forensics, but the species determination can be crucial, especially for samples where the morphology cannot easily be used to distinguish the donor organism (claws and bones of tigers and lions) or when the artifact tested belongs to so-called traditional Chinese medicine (tiger wine, broth, etc.) and species determination is required for legal reasons (endangered species can be listed in different CITES appendixes). The species determination from a sample of unknown origin can be performed using traditional methods like Sanger sequencing [47] or next-generation sequencing [48], mtDNA length polymorphisms [49,50], HRMA [51], or species-specific SNPs [52], but additional testing means more of a sample is consumed, more time is required, and more money is spent. Therefore, the species-specific markers included in the quantitation multiplex reaction make sense, even if a dual approach (quantitation targeting both tigers and lions using a specific probe/curve) would be better. DNA degradation does not necessarily need to be included when the nuclear target reflects the average size of STR amplicons used in multiplexes. The results obtained during the sensitivity testing for Pleo Qplex and Pleo STRplex are comparable to commercial kits for human DNA testing for identification purposes [53,54]. The set of genetic tools developed herein should be extended to address other scientific questions, like the animal’s geographic origin [55], Y-chromosomal [56] or mtDNA [57] lineages, visible traits [58], or the presence of hybrids [59]. Future research on additional genetic tools for endangered animals will require extensive collaboration on sample collection [60].

Inhibitors can be co-purified from various forensic samples [61], including animal and plant samples [62,63]. Thus, it is crucial to use extraction methods that remove the most inhibitors during the standard workflow. The remaining inhibitors, when detected during the quantitation step, can be removed by an additional cleaning step, e.g., using spin columns [64] or other methods like ultrafiltration [65], size exclusion chromatography [66], electro membrane extraction [67], dialysis [68], chemical treatment [69], or microfluidic devices [70].

The set of STRs comprising the Pleo STRplex and Ptig STRplex kits can be used for any genus of Panthera’s individuals. This makes these multiplexes universal and applicable to a broader spectrum of samples. However, as demonstrated on locus Pleo 32, not all species belonging to the same genus are equally polymorphic in all loci of the presented multiplex. Even STRs used in human DNA typing can be applied to samples of phylogenetically related species like primates [71,72,73]. Another limitation observed during the work with endangered species is the lack of a sufficient number of unrelated individuals to perform a population study and test the independence of the inheritance of the loci, to be able to calculate the paternity indexes, matching probabilities, and the combined power of exclusion (see Harper and De Bruyn). This problem can be solved by the creation of centralized databases run by reference laboratories like the RhODIS^®^ (The Rhinoceros DNA Index System) database in South Africa (see Harper). Several commercial software solutions, originally developed for human DNA typing, are suitable for non-human DNA databasing and kinship analysis (see DNA DATABASE MANAGEMENT, ENFSI DNA Working Group, [74]). The recently released GenoProof Suite (Qualitype, Dresden, Germany), used in this study, offers statistical analysis based on Bayesian mathematics and matching features. The limited amount of *P. leo* samples prevented us from performing a population study.

## 5. Conclusions

We developed a sensitive and species-specific qPCR quantitation system, Pleo Qplex, that enables the measurement of nuclear and mitochondrial DNA concentrations and the detection of copurified inhibitors. The STR multiplex, Pleo STRplex, extends the set of STR loci applicable to the individual identification of big cats. The results of this study already serve Czech and foreign law enforcement and environmental agencies. More typed and databased samples will solve the limitations of the lack of population studies for Panthera genus members.

## Figures and Tables

**Table 1:** Primers and probes used in *Pleo Qplex*.

Primer/Probe Name	Final Concentration (µM)	Sequence (5′-3′)	PCR Product Size (* bp)	Specificity	TaqMan Probe Fluorescent Label
qPleoM_f	0.75	ACCTATTAGGAGATCCCGACAAC	150	*cytB* (mtDNA)	---
qPleoM_r	0.75	CTGTTTGGAAGTGTGGAGGGCA			---
qPleoM_p	0.25	TACCCCCGCCAATCCTCTAAGCACC		probe	VIC
qPleoN_f	0.5	CGTTCTTGGAACGCTGCATA	~215–260	STR locus *Pati01* ** (nDNA)	---
qPleoN_r	0.5	ATGGGCAGCACTCGTATGAT			---
qPleoN_p	0.25	ATGCTACAGAAATAGAAGCCAA		probe	6-FAM
qPleoC_f	0.5	GAGACGAATACCAACCGGCA	366	*IPC* (Internal Positive Control)	---
qPleoC_r	0.5	GGACCATGCTTGCGTTTGAG			---
qPleoC_p	0.25	TCGACGATTCAAGCACGAT		probe	NED

* bp = base pairs; ** (Wu et al.) [40].

**Table 2:** The qPCR reaction setup.

qPCR Reaction Composition	10 µL Reaction	Final Concentration in qPCR
2× *TaqMan* Multiplex Master Mix	5 µL	1×
20× qPleo mtDNA Assay Mix (20× qPleoM)	0.5 µL	1×
20× qPleo nDNA Assay Mix (20× qPleoN)	0.5 µL	1×
20× qPleo IPC DNA Assay Mix (20× qPleoC)	0.5 µL	1×
IPC DNA (0.1 pg/μL)	1 µL	0.1 pg
Template DNA	1 µL	different
H_2_O	1.5 µL	

**Table 3:** STR multiplex *Pleo STRPlex*.

Name	Repeat Structure	Repetition	Size (bp *)	5’ Primer Fluorescent Label
Pleo24	(CA)n	2n	105–160	FAM
Pleo30	(ATGG)n (GATA)n (TAGA)n	4n	180–240	FAM
Pleo23	(ATGT)n (GT)n (AC)n (AT)n	2n	250–380	FAM
Pleo22	(TAGA)n	4n	105–168	ATTO550
Pleo31	(GATA)n (GA)n	4n	175–220	ATTO550
Pleo32	(TCTG)n (TCTA)n	4n	270–360	ATTO550
Pleo33	(CAGA)n (TAGA)n	4n	180–280	ATTO565

* bp = base pairs.

**Table 4:** Primer sequences *Pleo STRPlex*.

STR Marker	Primer Sequence 5′ → 3′
Pleo 24	F: GTGTAGTTATGTGTATTATGAATGTGTGTATGC
	R: AATATCTTAGCAGATGGAGCTGGG
Pleo 30	F: GGCCTTCTAACTTCCTTGCAGA
	R: CATTTAGTTAGCCCATTTTCATCA
Pleo 23	F: CTGTTTGACAGTACAAGTATTACTGGCC
	R: GGTCTATGTGTCTCTGTTTCCTCTTATG
Pleo 22	F: TAAGAATTTATGGATTACTCGGCAAAT
	R: TATTCATTGTAGTCCCTGGGATTG
Pleo 31	F: GAGTTAGGACAAGATTATCAAGGAACTTG
	R: CAGTCTGAGCTTAGAGTCTGCTCAAG
Pleo 32	F: GGGCAAATACACTAACCA
	R: CTCCTGCTAGAATCTCCAA
Pleo 33	F: TCTTTGTTTGGCTATAACCATTCACTAG
	R: AACCCAGTGTCTCCTTGTACCAC

**Table 5:** PCR set-up.

PCR	12.5 µL Reaction	Final Concentration in PCR
Gold Star 10× buffer	1.25 µL	1×
10× *Pleo STRPlex* Primer Mix	1.25 µL	1×
Template DNA	different	10 pg nuclear DNA
AmpliTaq Gold DNA polymerase	0.25 µL	2.5 U/PCR
H_2_O	to 12.5 µL	

**Table 6:** Pleo STRplex allelic ladder alleles.

STRs	Alelles
Pleo24	15, 16, 19, 20, 21, 22, 23
Pleo30	20, 21, 22, 23, 24, 25, 26, 27
Pleo23	50, 51, 52, 53, 5, 56, 62, 65, 66, 67
Pleo22	14, 14.1, 15, 16.1, 17, 18, 20
Pleo31	18, 19, 20, 21
Pleo32	11, 14, 15, 16, 17, 18, 19, 20
Pleo33	13, 15, 16, 17, 18

## Data Availability

The data presented in this study are available on request from the corresponding author due to request of the funding agency.

## References

[1] Brugière D., Chardonnet B., Scholte P. (2015). Large-scale extinction of large carnivores (lion *Panthera leo*, cheetah *Acinonyx jubatus* and wild dog *Lycaon pictus*) in protected areas of West and Central Africa. Trop. Conserv. Sci..

[2] Cazalis V., Santini L., Lucas P.M., González-Suárez M., Hoffmann M., Benítez-López A., Pacifici M., Schipper A.M., Böhm M., Zizka A. (2023). Prioritizing the reassessment of data-deficient species on the IUCN Red List. Conserv. Biol..

[3] Whitman K., Starfield A.M., Quadling H.S., Packer C. (2004). Sustainable trophy hunting of African lions. Nature.

[4] Johanisová L., Mauerhofer V. (2023). Assessing trophy hunting in South Africa by comparing hunting and exporting databases. J. Nat. Conserv..

[5] Everatt K., Kokes R., Lopez Pereira C. (2019). Evidence of a further emerging threat to lion conservation; targeted poaching for body parts. Biodivers. Conserv..

[6] Coals P.G., Mbongwa N.S., Naude V.N., Williams V.L. (2022). Contemporary cultural trade of lion body parts. Animals.

[7] Bauer H., Nowell K., Sillero-Zubiri C., Macdonald D.W. (2018). Lions in the modern arena of CITES. Conserv. Lett..

[8] Coals P., Moorhouse T.P., D’Cruze N.C., Macdonald D.W., Loveridge A.J. (2020). Preferences for lion and tiger bone wines amongst the urban public in China and Vietnam. J. Nat. Conserv..

[9] Sibanda L., van der Meer E., Johnson P.J., Hughes C., Dlodlo B., Parry R.H., Mathe L.J., Hunt J.E., Macdonald D.W., Loveridge A.J. (2021). Evaluating the effects of a conservation intervention on rural farmers’ attitudes toward lions. Hum. Dimens. Wildl..

[10] Vieira Da Silva C., Afonso Costa H., Costa Santos J., Espinheira R. (2012). Forensic Genetics as a Tool for Peace and Justice: An Overview on DNA Quantification. J. Forensic Res..

[11] Vajpayee K., Dash H.R., Parekh P.B., Shukla R.K. (2023). PCR Inhibitors and Facilitators-Their Role in Forensic DNA Analysis. Forensic Sci. Int..

[12] Kuffel A., Gray A., Daeid N.N. (2021). Impact of metal ions on PCR inhibition and RT-PCR efficiency. Int. J. Leg. Med..

[13] Hedman J., Rådström P. (2013). Overcoming inhibition in real-time diagnostic PCR. PCR Detection of Microbial Pathogens.

[14] Andréasson H., Gyllensten U., Allen M. (2002). Real-time DNA quantification of nuclear and mitochondrial DNA in forensic analysis. Biotechniques.

[15] Fregeau C.J., Fourney R.M. (1993). DNA typing with fluorescently tagged short tandem repeats: A sensitive and accurate approach to human identification. Biotechniques.

[16] Puri A. (2000). An international DNA database: Balancing hope, privacy, and scientific error. BC Int’l Comp. L. Rev..

[17] Khan R. (2021). Is the FBI’s Criminal Justice Database, CODIS, Approaching Its Expiration Date?. Forensic Genom..

[18] Bataille M., Crainic K., Leterreux M., Durigon M., de Mazancourt P. (1999). Multiplex amplification of mitochondrial DNA for human and species identification in forensic evaluation. Forensic Sci. Int..

[19] Hellmann A.P., Rohleder U., Eichmann C., Pfeiffer I., Parson W., Schleenbecker U. (2006). A proposal for standardization in forensic canine DNA typing: Allele nomenclature of six canine-specific STR loci. J. Forensic Sci..

[20] Kanthaswamy S. (2015). domestic animal forensic genetics–biological evidence, genetic markers, analytical approaches and challenges. Anim. Genet..

[21] Menotti-Raymond M., Stephens J., Lyons L., O’Brien S., David V. (1997). Genetic individualization of domestic cats using feline STR loci for forensic applications. J. Forensic Sci..

[22] Halverson J.L., Basten C. (2005). Forensic DNA identification of animal-derived trace evidence: Tools for linking victims and suspects. Croat. Med. J..

[23] Lorenzini R., Cabras P., Fanelli R., Carboni G.L. (2011). Wildlife molecular forensics: Identification of the Sardinian mouflon using STR profiling and the Bayesian assignment test. Forensic Sci. Int. Genet..

[24] Caratti S., Rossi L., Sona B., Origlia S., Viara S., Martano G., Torre C., Robino C. (2010). Analysis of 11 tetrameric STRs in wild boars for forensic purposes. Forensic Sci. Int. Genet..

[25] Harper C.K. (2021). RhODIS®(The Rhinoceros DNA Index System): The Application of Simple Forensic and Genetic Tools Help Conserve African Rhinoceros. Wildlife Biodiversity Conservation: Multidisciplinary and Forensic Approaches.

[26] Harper C., Ludwig A., Clarke A., Makgopela K., Yurchenko A., Guthrie A., Dobrynin P., Tamazian G., Emslie R., van Heerden M. (2018). Robust forensic matching of confiscated horns to individual poached African rhinoceros. Curr. Biol..

[27] Singh A., Priyambada P., Jabin G., Singh S.K., Joshi B.D., Venkatraman C., Chandra K., Sharma L.K., Thakur M. (2020). Pangolin Indexing System: Implications in forensic surveillance of large seizures. Int. J. Leg. Med..

[28] Roberto B., Mauro Z., Claudia C., Gianluca D., Marta B., Michel D., Luciano D.T., Luigi L.F., Oliviero O., Francesco P. (2018). Who’s who in the western Hermann’s tortoise conservation: A STR toolkit and reference database for wildlife forensic genetic analyses. bioRxiv.

[29] Biello R., Zampiglia M., Corti C., Deli G., Biaggini M., Crestanello B., Delaugerre M., Di Tizio L., Leonetti F.L., Casari S. (2021). Mapping the geographic origin of captive and confiscated Hermann’s tortoises: A genetic toolkit for conservation and forensic analyses. Forensic Sci. Int. Genet..

[30] Jan C., Fumagalli L. (2016). Polymorphic DNA microsatellite markers for forensic individual identification and parentage analyses of seven threatened species of parrots (family Psittacidae). PeerJ.

[31] Willows-Munro S., Kleinhans C. (2020). Testing microsatellite loci for individual identification of captive African grey parrots (Psittacus erithacus): A molecular tool for parentage analysis that will aid in monitoring legal trade. Conserv. Genet. Resour..

[32] De Bruyn M., Dalton D.L., Mwale M., Ehlers K., Kotze A. (2024). Development and Validation of a Novel Forensic STR Multiplex Assay for Blue (*Anthropoides paradiseus*), Wattled (*Bugeranus carunculatus*), and Grey-Crowned Crane (*Balearica regulorum*). Forensic Sci. Int. Genet..

[33] Potoczniak M.J., Chermak M., Quarino L., Tobe S.S., Conte J. (2020). Development of a multiplex, PCR-based genotyping assay for African and Asian elephants for forensic purposes. Int. J. Leg. Med..

[34] Kinuthia J., Harper C., Muya S., Kimwele C., Alakonya A., Muigai A., Gakuya F., Mwaniki M., Gatebe E. (2015). The selection of a standard STR panel for DNA profiling of the African elephant (*Loxodonta africana*) in Kenya. Conserv. Genet. Resour..

[35] Vaněk D., Ehler E., Vaňková L. (2021). Development of DNA quantitation and STR typing systems for Panthera tigris species determination and individual identification in forensic casework. Eur. J. Environ. Sci..

[36] Liu Y., Xu J., Chen M., Wang C., Li S. (2019). A unified STR profiling system across multiple species with whole genome sequencing data. BMC Bioinform..

[37] Dawnay N., Ogden R., Wetton J.H., Thorpe R.S., McEwing R. (2009). Genetic data from 28 STR loci for forensic individual identification and parentage analyses in 6 bird of prey species. Forensic Sci. Int. Genet..

[38] Olsson I.A.S., Silva S.P.d., Townend D., Sandøe P. (2017). Protecting animals and enabling research in the European Union: An overview of development and implementation of directive 2010/63/EU. ILAR J..

[39] Hebenstreitova K., Salaba O., Trubac J., Kufnerova J., Vanek D. (2024). The Influence of Tanning Chemical Agents on DNA Degradation: A Robust Procedure for the Analysis of Tanned Animal Hide—A Pilot Study. Life.

[40] Wu J.-H., Lei Y.-L., Fang S.-G., Wan Q.-H. (2009). Twenty-one novel tri-and tetranucleotide microsatellite loci for the Amur tiger (*Panthera tigris altaica*). Conserv. Genet..

[41] Swango K.L., Hudlow W.R., Timken M.D., Buoncristiani M.R. (2007). Developmental validation of a multiplex qPCR assay for assessing the quantity and quality of nuclear DNA in forensic samples. Forensic Sci. Int..

[42] Holt A., Wootton S.C., Mulero J.J., Brzoska P.M., Langit E., Green R.L. (2016). Developmental validation of the Quantifiler® HP and Trio Kits for human DNA quantification in forensic samples. Forensic Sci. Int. Genet..

[43] Holmes A.S., Houston R., Elwick K., Gangitano D., Hughes-Stamm S. (2018). Evaluation of four commercial quantitative real-time PCR kits with inhibited and degraded samples. Int. J. Leg. Med..

[44] Ewing M.M., Thompson J.M., McLaren R.S., Purpero V.M., Thomas K.J., Dobrowski P.A., DeGroot G.A., Romsos E.L., Storts D.R. (2016). Human DNA quantification and sample quality assessment: Developmental validation of the PowerQuantĘr) system. Forensic Sci. Int. Genet..

[45] Simoes Dutra Correa H., Brescia G., Cortellini V., Cerri N., Verzeletti A. (2020). DNA quantitation and degradation assessment: A quantitative PCR protocol designed for small forensic genetics laboratories. Electrophoresis.

[46] Pineda G.M., Montgomery A.H., Thompson R., Indest B., Carroll M., Sinha S.K. (2014). Development and validation of InnoQuant™, a sensitive human DNA quantitation and degradation assessment method for forensic samples using high copy number mobile elements Alu and SVA. Forensic Sci. Int. Genet..

[47] Dawnay N., Ogden R., McEwing R., Carvalho G.R., Thorpe R.S. (2007). Validation of the barcoding gene COI for use in forensic genetic species identification. Forensic Sci. Int..

[48] Galan M., Pagès M., Cosson J.-F. (2012). Next-generation sequencing for rodent barcoding: Species identification from fresh, degraded and environmental samples. PLoS ONE.

[49] Vankova L., Vanek D. (2024). Capillary-Electrophoresis-Based Species Barcoding of Big Cats: CR-mtDNA-Length Polymorphism. Life.

[50] Alves C., Pereira R., Prieto L., Aler M., Amaral C.R., Arévalo C., Berardi G., Di Rocco F., Caputo M., Carmona C.H. (2017). Species identification in forensic samples using the SPInDel approach: A GHEP-ISFG inter-laboratory collaborative exercise. Forensic Sci. Int. Genet..

[51] Dule E.J., Kinimi E., Bakari G.G., Max R.A., Lyimo C.M., Mushi J.R. (2024). Species authentication in meat products sold in Kilosa District in Tanzania using HRM-enhanced DNA barcoding. J. Consum. Prot. Food Saf..

[52] Chang M., Kim J.-Y., Lee H., Lee E.-J., Lee W.-H., Moon S., Choe S., Choung C.M. (2022). Development of diagnostic SNP markers and a novel SNP genotyping assay for distinguishing opium poppies. Forensic Sci. Int..

[53] Gouveia N., Brito P., Serra A., Balsa F., Andrade L., Bento M.S., Cunha P., Bogas V., Lopes V., Porto M. (2015). Validation of Quantifiler^®^ Trio DNA Quantification kit in forensic samples. Forensic Sci. Int. Genet. Suppl. Ser..

[54] Wang D.Y., Chang C.W., Lagacé R.E., Calandro L.M., Hennessy L.K. (2012). Developmental validation of the AmpFℓSTR® Identifiler® Plus PCR Amplification Kit: An established multiplex assay with improved performance. J. Forensic Sci..

[55] Ogden R., Linacre A. (2015). Wildlife forensic science: A review of genetic geographic origin assignment. Forensic Sci. Int. Genet..

[56] Wheeldon T.J., Rutledge L.Y., Patterson B.R., White B.N., Wilson P.J. (2013). Y-chromosome evidence supports asymmetric dog introgression into eastern coyotes. Ecol. Evol..

[57] Verscheure S., Backeljau T., Desmyter S. (2013). Reviewing population studies for forensic purposes: Dog mitochondrial DNA. ZooKeys.

[58] Berger C., Heinrich J., Berger B., Hecht W., Parson W., CaDNAP (2021). Towards forensic DNA phenotyping for predicting visible traits in dogs. Genes.

[59] Enenkel K.A. (2014). 2 The Species and Beyond: Classification and the Place of Hybrids in Early Modern Zoology. Zoology in Early Modern Culture: Intersections of Science, Theology, Philology, and Political and Religious Education.

[60] Pérez-Espona S., Consortium C. (2021). Conservation-focused biobanks: A valuable resource for wildlife DNA forensics. Forensic Sci. Int. Anim. Environ..

[61] Alaeddini R. (2012). Forensic implications of PCR inhibition—A review. Forensic Sci. Int. Genet..

[62] Ramón-Laca A., Soriano L., Gleeson D., Godoy J.A. (2015). A simple and effective method for obtaining mammal DNA from faeces. Wildl. Biol..

[63] Ruggieri J., Kemp R., Forman S., Van Eden M.E. (2016). Techniques for nucleic acid purification from plant, animal, and microbial samples. Sample Preparation Techniques for Soil, Plant, and Animal Samples.

[64] Yang D., Eng B., Dudar J., Saunders S., Waye J. (1997). Removal of PCR inhibitors using silica-based spin columns: Application to ancient bones. Can. Soc. Forensic Sci. J..

[65] Queiroz A.P.S., Santos F., Sassaroli A., Hársi C., Monezi T., Mehnert D. (2001). Electropositive filter membrane as an alternative for the elimination of PCR inhibitors from sewage and water samples. Appl. Environ. Microbiol..

[66] Matheson C.D., Marion T.E., Hayter S., Esau N., Fratpietro R., Vernon K.K. (2009). Removal of metal ion inhibition encountered during DNA extraction and amplification of copper-preserved archaeological bone using size exclusion chromatography. Am. J. Phys. Anthropol. Off. Publ. Am. Assoc. Phys. Anthropol..

[67] Liu C., Huang D., Yang L., Wu S., Shen X., Pedersen-Bjergaard S., Huang C. (2021). Removal of polymerase chain reaction inhibitors by electromembrane extraction. Anal. Chem..

[68] Votrubova-Dubska J., Vanek D., Zikmund J., Mestek O., Urbanova V., Brzobohata H., Brestovansky P. (2016). Efficient removal of a PCR inhibitory agent (vivianite) found on excavated bones. Forensic Sci. Int..

[69] Teng F., Guan Y., Zhu W. (2008). A simple and effective method to overcome the inhibition of Fe to PCR. J. Microbiol. Methods.

[70] Geng T., Mathies R.A. (2015). Minimizing inhibition of PCR-STR typing using digital agarose droplet microfluidics. Forensic Sci. Int. Genet..

[71] Minaguchi K., Takenaka O. (2000). Structural variations of the VWA locus in humans and comparison with non-human primates. Forensic Sci. Int..

[72] Crouse C.A., Schumm J. (1995). Investigation of species specificity using nine PCR-based human STR systems. J. Forensic Sci..

[73] Ely J.J., Gonzalez D.L., Reeves-Daniel A., Stone W.H. (1998). Individual identification and paternity determination in chimpanzees (*Pan troglodytes*) using human short tandem repeat (STR) markers. Int. J. Primatol..

[74] ENFSI D (2016). DNA Database Management. https://enfsi.eu/wp-content/uploads/2016/09/final_version_enfsi_2016_document_on_dna-database_management_0.pdf.

